# Transcriptome and proteome analysis of dogs with precursor targeted immune-mediated anemia treated with splenectomy

**DOI:** 10.1371/journal.pone.0285415

**Published:** 2023-05-05

**Authors:** Mei Sugawara-Suda, Keitaro Morishita, Osamu Ichii, Takashi Namba, Keisuke Aoshima, Yumiko Kagawa, Sangho Kim, Kenji Hosoya, Nozomu Yokoyama, Noboru Sasaki, Kensuke Nakamura, Jumpei Yamazaki, Mitsuyoshi Takiguchi

**Affiliations:** 1 Laboratory of Veterinary Internal Medicine, Department of Clinical Sciences, Faculty of Veterinary Medicine, Hokkaido University, Hokkaido, Japan; 2 Veterinary Teaching Hospital, Department of Veterinary Clinical Sciences, Faculty of Veterinary Medicine, Hokkaido University, Hokkaido, Japan; 3 Laboratory of Anatomy, Department of Basic Veterinary Sciences, Faculty of Veterinary Medicine, Hokkaido University, Hokkaido, Japan; 4 Laboratory of Comparative Pathology, Department of Clinical Sciences, Faculty of Veterinary Medicine, Hokkaido University, Sapporo, Hokkaido, Japan; 5 North Lab, Sapporo, Japan; 6 Laboratory of Veterinary Surgery, Department of Veterinary Clinical Sciences, Faculty of Veterinary Medicine, Hokkaido University, Sapporo, Japan; 7 Translational Research Unit, Veterinary Teaching Hospital, Faculty of Veterinary Medicine, Hokkaido University, Sapporo, Hokkaido, Japan; 8 One Health Research Center, Hokkaido University, Hokkaido, Japan; Universidade Federal de Minas Gerais, BRAZIL

## Abstract

Precursor-targeted immune-mediated anemia (PIMA) in dogs is characterized by persistent non-regenerative anemia and ineffective erythropoiesis, and it is suspected to be an immune-mediated disease. Most affected dogs respond to immunosuppressive therapies; however, some are resistant. In this study, we carried out splenectomy as an alternative therapy for refractory PIMA in dogs, and analyzed gene expression levels in the spleen of dogs with or without PIMA and in serum before and after splenectomy. A total of 1,385 genes were found to express differentially in the spleens from dogs with PIMA compared with healthy dogs by transcriptome analysis, of which 707 genes were up-regulated, including *S100A12*, *S100A8*, and *S100A9* that are linked directly to the innate immune system and have been characterized as endogenous damage-associated molecular patterns. Furthermore, immunohistochemistry confirmed that S100A8/A9 protein expression levels were significantly higher in dogs with PIMA compared with those in healthy dogs. A total of 22 proteins were found to express differentially between the serum samples collected before and after splenectomy by proteome analysis, of which 12 proteins were up-regulated in the samples before. The lectin pathway of complement activation was identified by pathway analysis in pre-splenectomy samples. We speculated that S100A8/9 expression may be increased in the spleen of dogs with PIMA, resulting in activation of the lectin pathway before splenectomy. These findings further our understanding of the pathology and mechanisms of splenectomy for PIMA.

## Introduction

Non-regenerative immune-mediated anemia (NRIMA) [1, 2] and precursor targeted immune-mediated anemia (PIMA) [3–7] have been described in dogs with non-regenerative anemia and evidence of ineffective erythropoiesis. There are currently no clear criteria for differentiating between these two disease conditions, and both diseases show similar responses against immunosuppressive therapy. Recent studies of PIMA examined dogs with few signs of peripheral immune-mediated destruction (spherocyte 3%, positive saline agglutination test 1.5% [4]). NRIMA is considered to be a broader disease, and studies looking at NRIMA often include dogs with symptoms of immune-mediated hemolytic anemia (IMHA) (spherocyte 19%, positive saline agglutination test 54% [2]). Terminology and diagnostic criteria often vary between reports: in this study we use the term PIMA according to the inclusion criteria previously reported [3–8], since the dogs in this study did not have any concurrent IMHA symptoms. With reference to treatments, 50%–88% of dogs with NRIMA/PIMA respond to immunosuppressive therapies, some dogs fail to respond to the treatments [1–4]. Intriguingly, splenectomy has been recommended as an alternative treatment for refractory immune-mediated hematopoietic diseases in humans and dogs [8–12]. We previously found that 75% of dogs refractory to immunosuppressive therapy responded to splenectomy [13]. Spleen-specific plasma cells producing anti-platelet antibodies were reported to be responsible for the improvement in immune-mediated thrombocytopenia after splenectomy in humans; however, there have been no similar veterinary studies to determine the mechanism of splenectomy in animals [14]. We therefore carried out transcriptome analysis of the spleen and proteomic analysis of pre- and post-splenectomy serum samples from dogs with PIMA, to clarify the mechanisms responsible for the beneficial effects of splenectomy in dogs with PIMA.

## Materials and methods

### Ethics statement

Spleen and serum samples were obtained from client-owned dogs for veterinary diagnostic and treatment purposes with informed consent and the permission of the Ethics Screening Committee, Veterinary Teaching Hospital, Hokkaido University (permission number 2022–003). As controls, we used spleen samples that had been collected and preserved from healthy dogs that were euthanized in another study approved by the Hokkaido University Faculty of Veterinary Medicine Institutional Animal Care and Use Committee (#20–0081).

### Study population

This study included 21 client-owned dogs diagnosed with PIMA that were presented to the Department of Internal Medicine at the Veterinary Teaching Hospital of Hokkaido University. The inclusion criteria were as follows: (1) minimum 5-day history of severe non-regenerative anemia (hematocrit <30%) with an absolute reticulocyte count <60×10^3^/μL [1, 15]; (2) bone marrow cytology and/or histopathology results available with a diagnosis of ineffective erythropoiesis, defined as the presence of an erythropoietic response pattern (i.e., erythroid hypercellularity or increase in early-stage erythroid precursors with maturation arrest) not attributable to a pre-regenerative response or other disease process (e.g., myelodysplastic disease or drug reaction); (3) complete blood count, serum biochemistry profile, and thoracic and abdominal imaging results revealing no underlying cause for severe non-regenerative anemia; and (4) splenectomy performed because the animals had poorly responded to immunosuppressive therapy for >2 months, or because the animals were unable to receive immunosuppressive therapy due to suspected infection.

We also included three healthy dogs and three dogs without PIMA that underwent splenectomy because of a primary complaint of a spleen mass. Details of all the dogs are summarized in S1 Table.

### Spleen samples

The histopathology of the spleen samples was evaluated by a member of the American College of Veterinary Pathologists (YK). Spleens from the healthy control dogs showed no significant changes, but 3 non-PIMA and 21 PIMA spleen samples showed extramedullary hematopoiesis. Splenic masses were diagnosed as hematoma and adipose nodules. Spleen samples were collected and stored in 10% neutral buffered formalin or RNAlater (Qiagen, Hilden, Germany) buffer at −80°C for further histopathological and molecular analyses.

### Serum samples

Serum samples were collected only from dogs with PIMA. Blood samples were collected before and after splenectomy by jugular venipuncture into tubes containing a coagulation activator and a gel separator. The tubes were then kept at room temperature until visible clotting had occurred. The samples were then centrifuged at 1,000 × g for 10 min and the serum was separated and stored at −80°C until analysis. Pre-splenectomy serum samples were collected before blood transfusion. Post-splenectomy serum samples were collected approximately 2 months after surgery to eliminate the invasive effects of surgery, which may have affected the results.

### Transcriptome analysis

Total RNA was extracted from PIMA and normal spleen tissues using an RNeasy Mini Kit (Qiagen). RNA integrity was examined using an Agilent 2100 Bioanalyzer (Agilent Technologies Japan, Tokyo, Japan), and the RNA integrity number of the total RNA isolated from each spleen was >8.5. Sequencing libraries were prepared using a QuantSeq 3′mRNA-Seq Library Prep Kit for Illumina (FWD) (Lexogen GmbH, Wien, Austria). RNA sequencing (75-bp single-end) was conducted with NextSeq 500 (Illumina, San Diego, CA, USA) using a NextSeq 500/550 High Output Kit v2.5 Kit (Illumina), and a minimum of 2.1 million reads were generated for each sample. Quantified read counts and differentially expressed genes (DEGs) were determined using RaNa-Seq (a bioinformatics tool for the analysis of RNA-seq data; https://ranaseq.eu/index.php). FASTQ files were pre-processed with the Fastp tool [16], and expression was quantified using Salmon [17] with reference genome using Can_Fam_3.1. RNA sequences have been deposited in the repository of the DNA Data Bank of Japan with the accession number DRA015678. Differential expression was analyzed using DESeq2, and DEGs were determined based on an adjusted *p*-value of < 0.05 and log2 fold change (FC) > |2.0|. The comparison of distinct gene expression patterns was visualized in a principal component analysis (PCA) and volcano plots using R [18] and RJSplot [19].

### Immunohistochemistry (IHC) of the spleen

IHC was performed using deparaffinized histological sections (3 μm thickness). The sections were incubated in 10 mM citrate buffer (pH 6.0) for 15 min at 110°C, treated with 0.3% H_2_O_2_/methanol solution for 20 min, and blocked using 10% normal goat serum (SABPO kit; Nichirei Bioscience, Tokyo, Japan). The sections were then incubated overnight with primary antibodies to S100A8/A9 (1:5000; NBP2-45295; Novus Biologicals, Centennial, Co, USA.) at 4°C, followed sequentially by incubation with biotinylated goat anti-mouse IgG(H+L) (1:100;1031–08, Southern Biotech, Birmingham, AL, USA) for 30 min at room temperature, incubation with streptavidin-horseradish peroxidase using the SABPO kit (Nichirei Bioscience) for 30 min, and incubation with 3,3-diaminobenzidine tetrahydrochloride. Finally, the sections were counterstained with hematoxylin and examined under a microscope (Olympus, Tokyo, Japan), and converted to virtual slides using a Nano-Zoomer 2.0-RS (Hamamatsu Photonics, Shizuoka, Japan) at 40× magnification.

### Quantitative analysis for IHC samples

S100A8/A9 stained slides were analyzed with QuPath ver 3.2.0 [20]. Three 1 mm^2^ areas containing one lymph nodule were selected in each slide for S100A8/A9 signal counting. Hematoma and other lesions disrupting normal splenic structures were excluded from the analysis. To avoid the edge effects, areas within 500 μm from the tissue edge were not used. Cell segmentation was performed using StarDist extension for QuPath based on optical sum density values [21]. S100A8/A9 positive signals were distinguished from hemosiderin and formalin pigments at annotation steps. Annotation was performed every slide. The average number of positive cells per 1 mm^2^ area were used for further analyses.

### Serum proteome analysis

Albumin was removed from eight serum samples using a ProMax albumin removal kit (Bangs Laboratories Inc., Fishers, IN, USA). Ten microliters of serum was added to 35 μL of ProMax Binding/Wash Buffer and 50 μL of ProMax particles, followed by gentle mixing for 20 min at room temperature. The particles were then collected by magnetic separation and the supernatant containing albumin was removed. The particles were washed three times with 500 μL ProMax Binding/Wash Buffer and then mixed for 10 min at room temperature in 80 μL of 100 mM Tris-HCl pH 8.5 and 0.5% sodium dodecanoate. The supernatant was transferred to a fresh 1.5 mL tube after magnetic separation of the particles, 20 μL of the sample was treated with 10 mM dithiothreitol at 50°C for 30 min, followed by alkylation with 30 mM iodoacetamide in the dark at room temperature for 30 min. The reaction was stopped with 60 mM cysteine for 10 min. The mixture was then diluted with 150 μL of 50 mM Tris-HCl pH 8.0 and digested by adding 400 ng of Trypsin/Lys-C mix (Promega, Madison, WI, USA) overnight at 37°C. The digested sample was acidified with 30 μL of 5% trifluoroacetic acid followed by sonication (Bioruptor UCD-200, CosmoBio, Tokyo, Japan) for 5 min. The mixture was shaken for 5 min and centrifuged at 15,000 × *g* for 5 min and the supernatant was desalted using C18-StageTips [22], followed by drying using a centrifugal evaporator. The dried peptides were redissolved in 3% acetonitrile and 0.1% formic acid measured using a colorimetric peptide assay kit (Thermo Fisher Scientific, Waltham, MA, USA), and transferred to a hydrophilic-coated low-adsorption vial (ProteoSave vial; AMR Inc., Tokyo, Japan).

For liquid chromatography (LC) separation, the mobile phases consisted of 0.1% (v/v) formic acid as solvent A and 0.1% (v/v) formic acid/80% (v/v) acetonitrile as solvent B. Each peptide sample (200 ng) was injected directly onto a 75 μm × 12 cm nanoLC nano-capillary column (Nikkyo Technos Co., Ltd., Tokyo, Japan) at 40°C and then separated with a 40 min gradient at a flow rate of 150 nL/min using an UltiMate 3000 RSLCnano LC system (Thermo Fisher Scientific). Peptides eluted from the column were analyzed using a Q Exactive HF-X (Thermo Fisher Scientific) for overlapping window DIA-MS [23, 24]. Mass spectrometry (MS) 1 spectra were collected in the range of 495–785 m/z at 30,000 resolution to set an automatic gain control target of 3e6 and maximum injection time of 55. MS2 spectra were collected in the range >200 m/z at 15,000 resolution to set an automatic gain control target of 3e6, maximum injection time of “auto”, and a normalized collision energy of 28%. The isolation width for MS2 was set to 4 m/z and overlapping window patterns in 500–780 m/z were used window placements optimized by Skyline v4.1 [25].

MS files were searched against a dog spectral library using Scaffold DIA (Proteome Software, Inc., Portland, OR, USA). The dog spectral library was generated from dog protein sequence database (Proteome ID: UP000002254) by Prosit [26, 27]. The Scaffold DIA search parameters were as follows: experimental data search enzyme, trypsin; maximum missed cleavage sites, 1; precursor mass tolerance, 8 ppm; fragment mass tolerance, 8 ppm; and static modification, cysteine carbamidomethylation. The protein identification threshold was set for both peptide and protein false discovery rates (FDRs) of <1%. Peptide quantification was calculated using the EncyclopeDIA algorithm [28] in Scaffold DIA. For each peptide, the four highest quality fragment ions were selected for quantitation. Protein quantification was estimated from the summed peptide quantification. We only used proteins that met the criteria of a peptide FDR <1%, protein FDR <1%, and number of peptide fragments ≥2. The proteomic datasets have been deposited into the repository of ProteomeXchange and Japan ProteOme STandard Repository with the accession numbers PXD039913 and JPST002024 respectively. The thresholds for altered proteins were *p* < 0.05 (*t*-test) and fold change >2 or <0.5 between the pre- and post- splenectomy groups.

### Pathway analysis

DEG- and differentially expressed protein-related pathways were analyzed with PANTHER 17.0 (http://www.pantherdb.org/), using the annotation dataset “Reactome pathway” to identify the activated pathways. We only included significantly (*p* < 0.05) up- or down-regulated pathways in our results.

### Statistical analysis

Statistical analysis of the IHC results was performed using JMP Pro 16.0.0 (SAS Institute, Cary, NC, USA). The Steel–Dwass test was used to compare positive cells in PIMA spleens, non-PIMA spleens and healthy spleens in IHC slides. Statistical significance was defined as *p* < 0.05. Statistical analysis of the proteome results was performed using Microsoft Excel for Mac 16.64 (Microsoft Corporation, Redmond, WA, USA).

## Results

### Transcriptome analysis and pathway analysis

**Fig 1:**
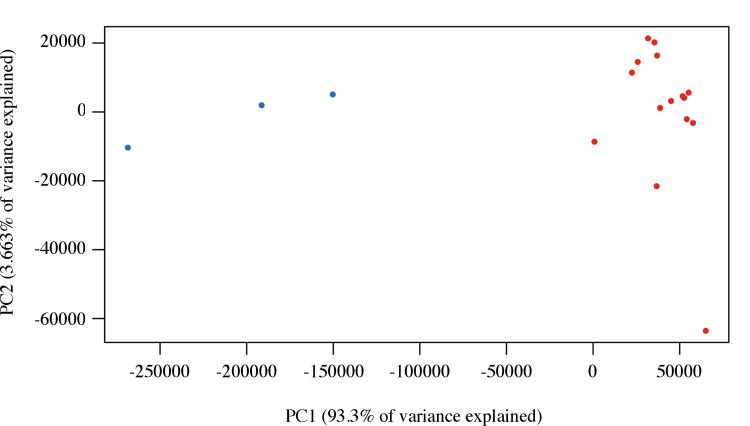
PCAs of spleen samples from dogs with PIMA and normal dogs. Red: dogs with PIMA; blue: healthy dogs.

We analyzed spleen samples from 15 dogs with PIMA and three healthy dogs to identify PIMA-related DEGs in the spleen. A total of 15,903 genes were detected by transcriptome analysis. The gene expression profiles were analyzed by PCA (Fig 1), which clearly separated normal spleen from PIMA samples.

**Fig 2:**
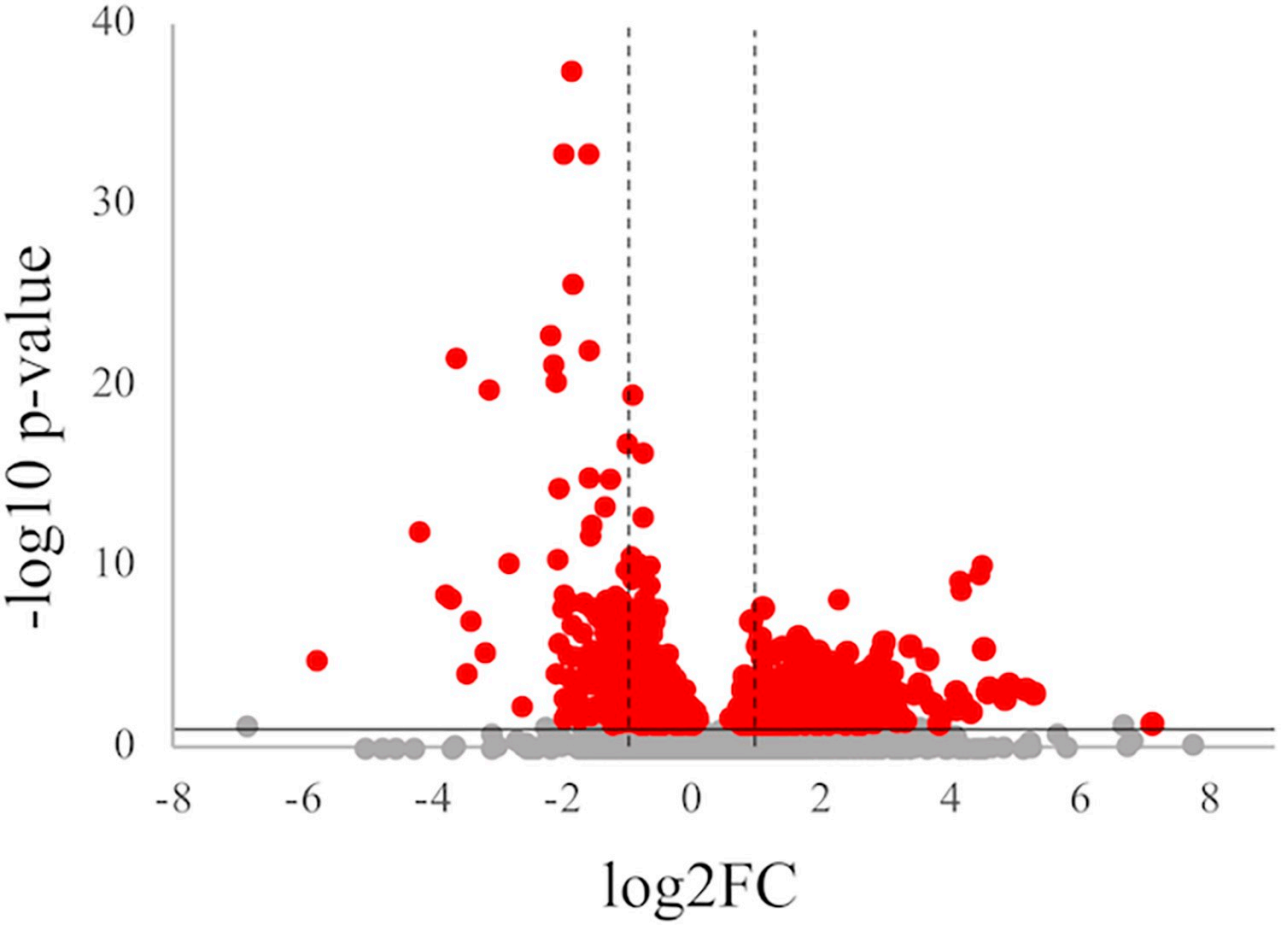
Volcano plot of DEGs detected by transcriptome analysis in dogs with PIMA versus healthy dogs. Red points indicate genes that were significantly increased or decreased in dogs with PIMA compared with healthy dogs (*p* < 0.05). The x-axis shows log2 fold-changes in expression and the y-axis shows the −log 10 *p*-value of a gene being differentially expressed. The two dotted lines show the size cut-offs for log2 fold-changes while the horizontal line shows the *p*-value cutoff.

We identified a total of 1,385 DEGs between spleen samples from normal dogs and dogs with PIMA (8.7% of all identified genes), with a *p-*value < 0.05. Of these, 707 genes were up-regulated and 678 were down-regulated in dogs with PIMA (Fig 2). The top 10 over- and under- expressed genes are shown in Table 1, and all DEGs are shown in S2 Table. We then performed pathway analysis of the 300 most strongly up- and down-regulated DEGs. The top 10 pathways enriched in the up-regulated genes were included in “Cell Cycle” and “DNA Replication”, possibly because extramedullary hematopoiesis was detected in all PIMA dogs but not in the normal dogs by histopathologic examination. The top 10 over- and under-expressed pathways are shown in Table 2 and S3 Table.

**Table 1:** Top 10 over- and under-expressed genes in dogs with PIMA vs healthy dogs.

Ensembl ID	Gene Symbol	Mean Expression	log2 Fold Change	adjusted *p*-value
ENSCAFG00000023324.2	S100A12	8755.88	4.49	9.60E-11
ENSCAFG00000017557.2	S100A8	8888.91	4.44	2.90E-10
ENSCAFG00000029470.1	S100A9	839.68	4.15	6.80E-10
ENSCAFG00000013763.3	LTF	192.64	4.16	2.30E-09
ENSCAFG00000007851.3	HMGB2	327.60	2.28	7.20E-09
ENSCAFG00000009488.5	MTDH	546.70	1.09	1.80E-08
ENSCAFG00000012057.4	EIF3A	126.76	0.91	1.00E-07
ENSCAFG00000006593.3	CBX5	227.34	1.64	7.30E-07
ENSCAFG00000012528.3	PSMA7	609.24	1.05	8.70E-07
ENSCAFG00000001211.2	HMGA1	47.22	2.97	1.50E-06
ENSCAFG00000011677.4	EPB41	86.04	-1.84	4.00E-38
ENSCAFG00000007478.3	CHMP4B	75.71	-1.59	1.60E-33
ENSCAFG00000006164.4	CYP1B1	315.00	-1.96	1.60E-33
ENSCAFG00000002513.4	TMEM196	24.76	-1.82	2.30E-26
ENSCAFG00000016920.4	ABI3	5.02	-2.17	1.70E-23
ENSCAFG00000010377.4	ENSCAFT00000048356.2	39.12	-1.60	9.10E-23
ENSCAFG00000000364.3	WIF1	4.33	-3.65	2.70E-22
ENSCAFG00000008756.3	ACTG2	19.44	-2.15	6.70E-22
ENSCAFG00000017326.3	CNN1	41.78	-2.10	5.10E-21
ENSCAFG00000018078.4	ENSCAFT00000028730.4	16.39	-3.13	1.40E-20

**Table 2:** Top 10 up- or down-regulated pathways of DEGs in dogs with PIMA.

Pathway name	Fold enrichment	Raw *p*-value	regulate	No. of DEG
Unclassified	0.57	8.81E-45	up	245
Cell Cycle	6.05	1.86E-43	up	100
Cell Cycle, Mitotic	6.31	1.14E-41	up	93
Cell Cycle Checkpoints	8.35	4.65E-31	up	57
M Phase	5.94	2.56E-28	up	66
Mitotic Anaphase	7.73	1.43E-26	up	51
Mitotic Metaphase and Anaphase	7.69	1.74E-26	up	51
Separation of Sister Chromatids	8.24	1.44E-25	up	47
Metabolism of RNA	4.17	1.60E-23	up	75
S Phase	8.85	6.73E-23	up	40
Signal Transduction	1.92	4.41E-11	down	114
RHO GTPase cycle	2.96	1.34E-08	down	38
Smooth Muscle Contraction	11.8	1.05E-07	down	10
RAC1 GTPase cycle	3.78	7.33E-07	down	21
Signaling by Rho GTPases	2.35	7.35E-07	down	43
Signaling by Rho GTPases, Miro GTPases and RHOBTB3	2.3	1.57E-06	down	43
Muscle contraction	3.9	3.01E-06	down	18
CDC42 GTPase cycle	3.8	4.23E-06	down	18
Cell Cycle	0.06	4.61E-06	down	1
Cell Cycle, Mitotic	0.07	2.09E-05	down	1

### IHC for S100A8/A9

**Fig 3:**
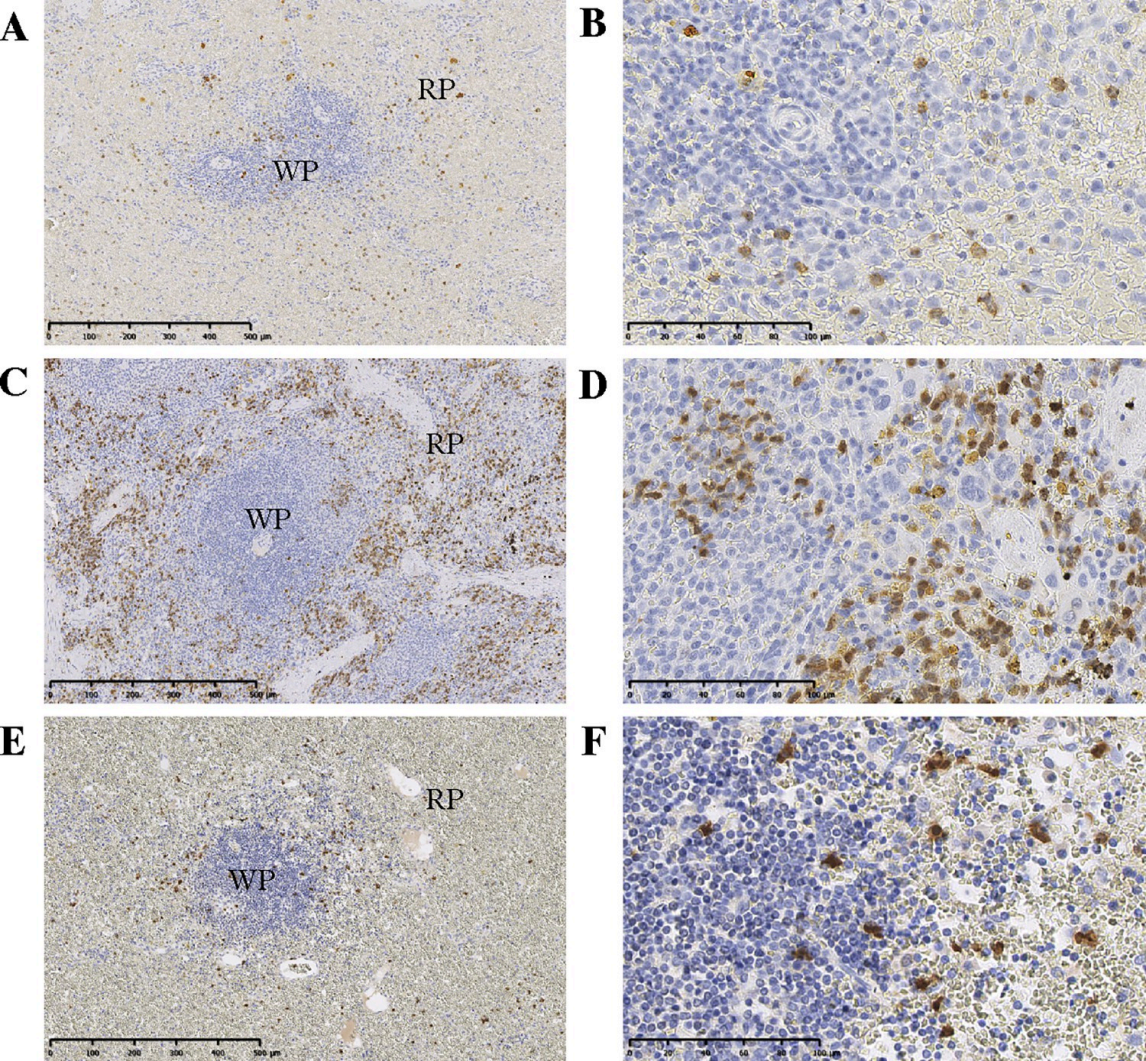
IHC analysis of S100A8/A9 in spleen samples from dogs with and without PIMA. Representative positive staining in a healthy dog (A, B), dog with PIMA (C, D), and dog with non-PIMA (E, F). RP = red pulp (in red), WP = white pulp (in blue). S100A8/A9 stained-cell (shown in brown). Scale bars = 500μm (A, C, E) and 100 μm (B, D, F).

**Fig 4:**
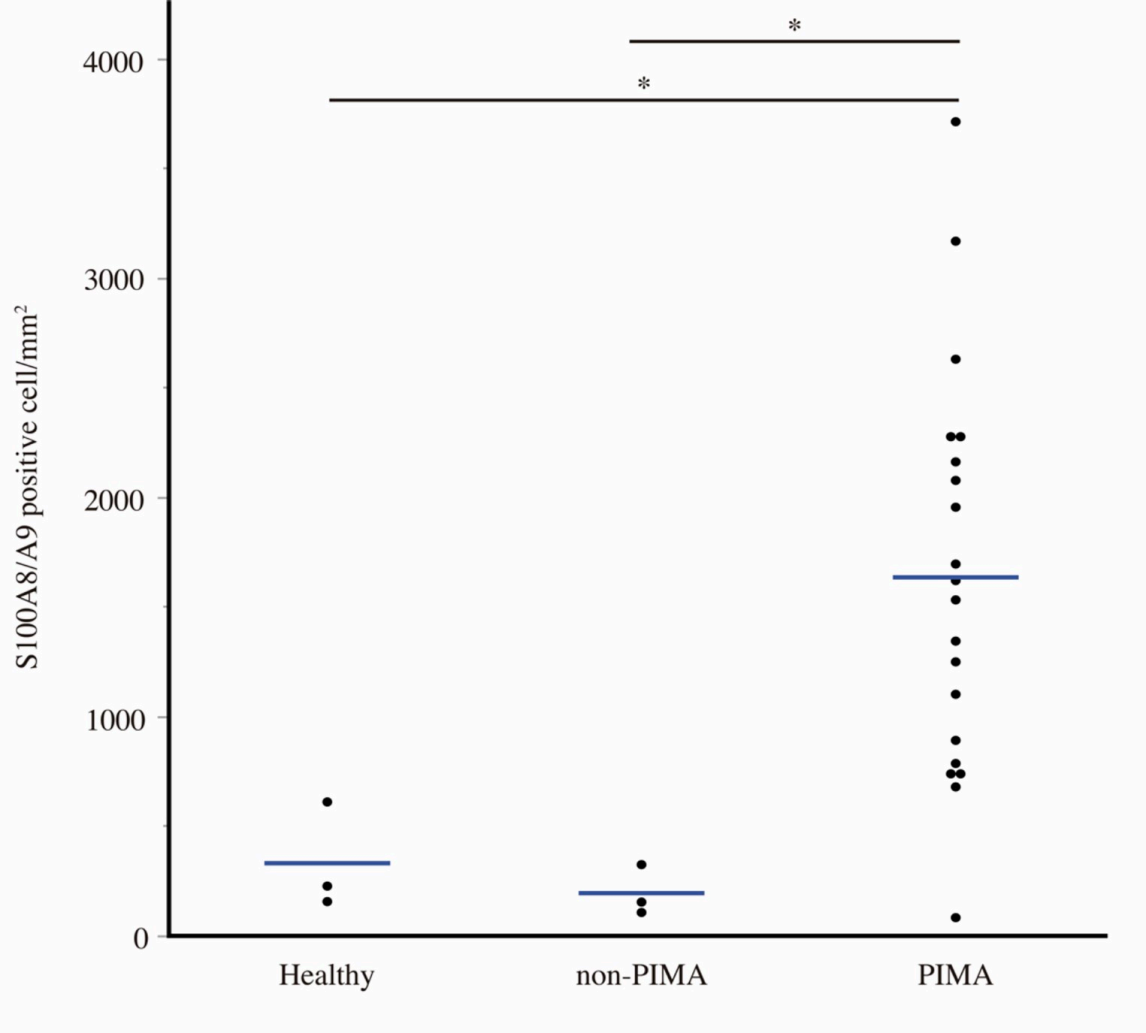
Numbers of S100A8/A9-positive cells in the spleen assessed by IHC. Dot plots represent mean number of S100A8/A9-positive cells per mm^2^ for each spleen sample. Statistical analysis performed using the Steel–Dwass test. *p < 0.05.

Transcriptome analysis revealed that *S100A12*, *S100A8*, and *S100A9* were the top over-expressed genes in PIMA spleen (*p* < 9.6E-11, 2.9E-10, and 6.8 E-10; Table 1). We specifically focused on *S100A8/A9*, which are also known to play a role in erythrocyte hematopoiesis and complement activation [29, 30]. We verified the protein expression levels and localization of S100A8/A9 in spleen samples from 21 dogs with PIMA, three healthy dogs, and three non-PIMA dogs, to investigate the relationship between S100A8/A9 and extramedullary hematopoiesis. Few S100A8/A9-positive cells were observed at the white pulp margin in healthy dogs and non-PIMA dogs, while numerous positive cells were observed in the medullary red pulp as well as the white pulp in dogs with PIMA (Fig 3). The number of S100A8/A9-positive cells was significantly higher in PIMA spleen compared with healthy and non-PIMA spleen samples (*p* = 0.0412 and *p* = 0.0412, respectively) (Fig 4).

### Differentially expressed proteins and pathways in pre- and post-splenectomy serum samples in dogs with PIMA

We performed proteomic profiling of serum samples using LC tandem mass spectrometry to reveal the physiological changes between pre- and post-splenectomy. We obtained pre- and post-splenectomy samples from four dogs with PIMA (8 samples) and identified 432 proteins in the eight serum samples, of which 342 proteins met the analysis criteria (S4 Table). A total of 22 proteins were differentially expressed between the pre- and post-splenectomy serum samples (*p* < 0.05, fold change ≥2) (Table 3). Twelve proteins were down-regulated and 11 were up-regulated in the post-splenectomy compared with the pre-splenectomy samples.

**Table 3:** Over- and under-expressed proteins in pre- and post-splenectomy serum samples from dogs with PIMA.

Uniprot Ac.	Protein name	Gene name	p value	ratio >2 or ratio <0.5
F1PPL0	BPI fold containing family B member 4	BPIFB4	3.76E-02	26.99
P19006	Haptoglobin	HP	5.07E-03	6.54
J9P7F7	Ficolin 1	FCN1	3.16E-02	4.00
F1PH87	Serpin family A member 3	SERPINA3	2.97E-02	3.60
F6USM4	Mannan binding lectin serine peptidase 2	MASP2	4.48E-02	2.80
Q28260	Vascular cell adhesion protein 1	VCAM1	6.49E-03	2.59
E2RNL2	Insulin like growth factor binding protein 7	IGFBP7	1.68E-04	2.58
F1PCE5	Serpin family A member 1	SERPINA1	4.62E-02	2.21
J9P9J6	Uncharacterized protein	-	1.64E-02	2.21
A0A5F4DC46	Endostatin domain-containing protein	-	3.02E-03	2.14
E2RLS3	Heat shock protein 90 alpha family class B member 1	HSP90AB1	4.64E-02	2.10
F1PGM9	Complement component 4 binding protein alpha	C4BPA	2.34E-02	0.48
A0A5F4C3Y4	Sex hormone binding globulin	SHBG	4.48E-02	0.45
A0A5F4D8N3	Lymphocyte cytosolic protein 1	LCP1	4.18E-02	0.41
A0A5F4BYK6	Tenascin C	TNC	2.37E-02	0.37
E2RJY0	Potassium channel tetramerization domain containing 12	KCTD12	1.44E-03	0.31
A0A5F4BVB4	ANTXR cell adhesion molecule 1	ANTXR1	1.20E-02	0.29
A0A5F4BTK2	Anthrax toxin receptor	ANTXR2	2.66E-02	0.28
A0A5F4CN27	Periostin	POSTN	1.11E-02	0.26
A0A5F4CTD0	Alpha-1-B glycoprotein	A1BG	1.14E-02	0.25
A0A5F4D9Z3	Serpin family A member 7	SERPINA7	1.38E-02	0.23
E2RNR9	Osteomodulin	OMD	3.93E-02	0.21

Finally, we focused on the enriched pathways related to 22 significantly altered serum proteins. The Reactome pathway “ficolins bind to repetitive carbohydrate structures on the target cell surface” was significantly up-regulated in pre-splenectomy samples (*p* = 4.91E-06) (S5 Table). This pathway included ficolin 1 (FCN1) and mannan-binding lectin serine peptidase 2 (MASP2), which were up-regulated 4- and 2.8-fold, respectively, in pre- compared with post-splenectomy samples.

## Discussion

In this study, we investigated the mechanisms responsible for the effects of splenectomy in dogs with PIMA by transcriptome analysis of spleen samples and proteomics analysis of pre- and post-splenectomy serum samples. Based on the results of transcriptome analysis, we validated the protein expression of S100A8/A9 in spleen samples using IHC.

In the transcriptome analysis, PCA of 15,903 genes showed a clear separation between spleen samples from dogs with PIMA and healthy dogs, suggesting that PIMA may significantly affect gene expression in the spleen. Among these, 1,385 genes were detected as DEGs, of which the top three most highly overexpressed genes in PIMA spleens were *S100A12*, *S100A8*, and *S100A9*. S100A8/A9 protein was also highly expressed, especially in PIMA samples, as shown by IHC. The S100 protein family comprises the largest subgroup within the Ca^2+^-binding EF-hand protein superfamily [31]. S100A8/A9 and S100A12 are released from activated monocytes and granulocytes and act as proinflammatory endogenous Toll-like receptor 4-ligands [32–34]. Serum levels of S100A12 and S100A8/A9 were shown to be increased in various inflammatory and autoimmune diseases, and complement activation was reported to occur at sites expressing S100A8/A9 [30, 31, 35–37]. Previous studies have suggested that PIMA may be an autoimmune disease [6], which may partially explain the elevated S1008/A9 levels.

One of the significant clinicopathological findings is the presence of ineffective erythropoiesis in dogs with PIMA. S100A9 was also shown to suppress erythroid differentiation in both experimentally induced deletion 5q subtype of myelodysplastic syndrome through inactivation of Rps14 and wild-type mouse model, specifically the hematopoietic stem cells and progenitor cells [29]. Moreover, serum levels of S100A8 were significantly elevated in patients with myelodysplastic syndrome compared with healthy controls [38]. Thus, the inhibitory effect of S100A9 on erythroblast differentiation may be responsible for the ineffective erythropoiesis in PIMA dogs.

We also carried out proteomic analysis of pre- and post-splenectomy serum samples and found that 22 proteins were differentially expressed. Bactericidal/permeability-increasing fold-containing family B member 4 (BPIFB4), haptoglobin, and FCN1 were specifically down-regulated in post-splenectomy compared with pre-splenectomy serum samples. BPIFB4 serum levels were 26-fold higher before compared with after splenectomy, while its RNA expression levels were significantly higher in PIMA spleens than those in healthy spleens (*p* = 0.003). Given these results, BPIFB4 may be produced in the spleen, and may be expressed at higher levels in dogs with PIMA. The concentration of BPIFB4 in serum appears to then decrease after a splenectomy. However, these data are inconsistent with the fact that BPIFB4 is typically more abundant in the serum of healthy, long-lived individuals compared with frail individuals [39]. This has been postulated to be due to advantageous anti-inflammatory effects through macrophage polarization [40] and correction of hypertension in humans [41]. Unfortunately, there is insufficient information to understand the direct involvement of BPIFB4 in non-regenerative anemia, and its association with the pathogenesis of PIMA remains unknown.

A study that investigated the clinical progression of horses after splenectomy found that two months after the procedure, the concentration of haptoglobin was significantly reduced compared with those that had not been splenectomized [42]. Our findings are consistent with these data, and the collection timelines were similar because we collected post-splenectomy serum samples from the dogs more than 2 months after the procedure. However, haptoglobin levels can be influenced by the administration of corticosteroids to dogs, and increased haptoglobin levels are often observed after corticosteroid therapy [43, 44] and in spontaneous hyperadrenocorticism [45]. Together, these data suggest that changes in serum haptoglobin could be affected by the splenectomy; however, we cannot exclude the possibility of elevated haptoglobin due to corticosteroid therapy.

**Fig 5:**
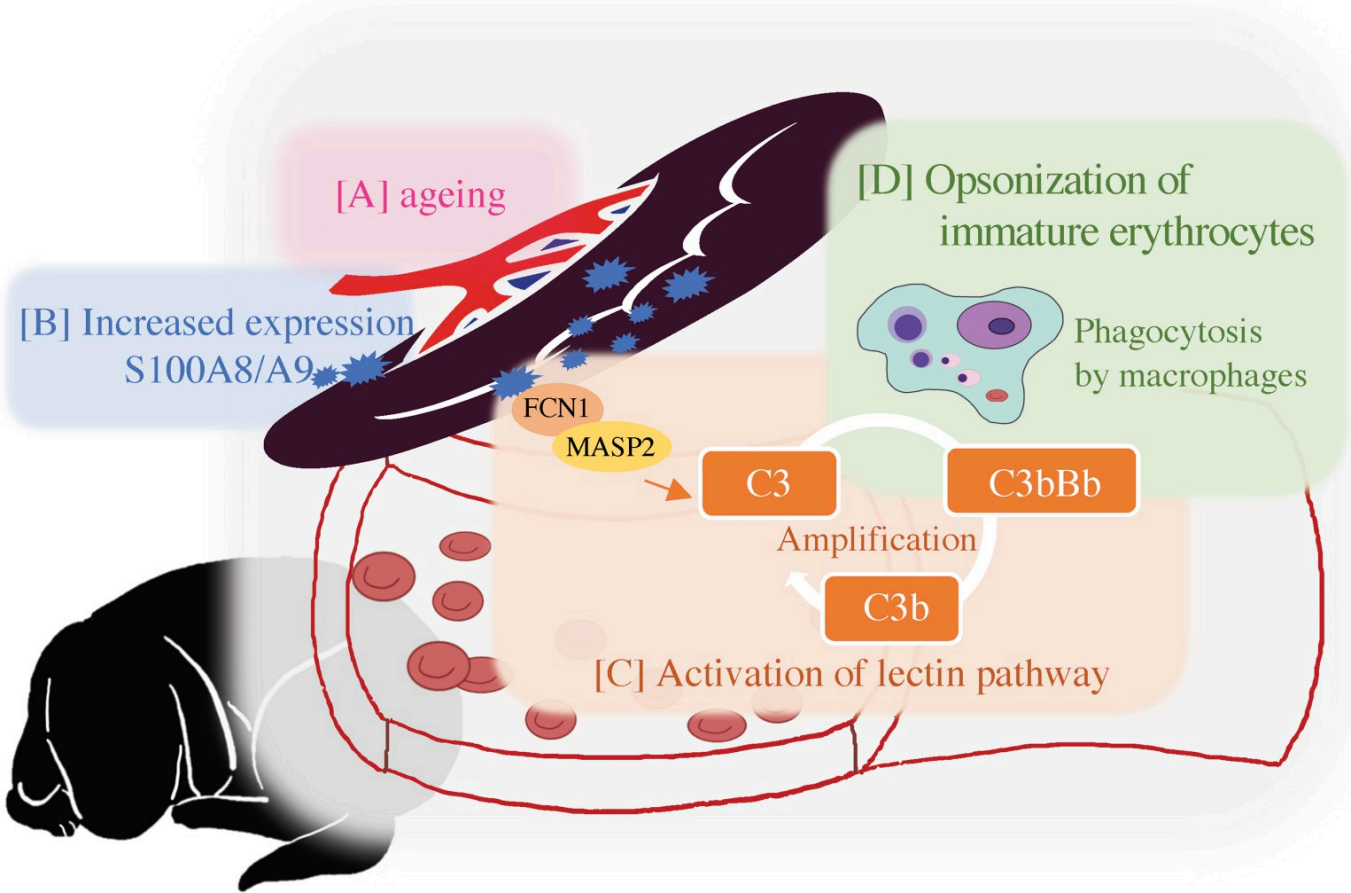
Schematic diagrams illustrating possible mechanisms underlying PIMA in dogs. [A], [B] Age-related changes in the spleen lead to increased expression of S100A8/A9. [C] S100A8/A9 promotes FCN1 recognition and MASP2 regulation, which activates the lectin complement pathway. [D] The lectin complement pathway triggers opsonization of immature erythrocytes and phagocytosis by macrophages.

The reactome pathways “Lectin pathway of complement activation” were over-represented in our list of pathways upregulated before splenectomy. The complement system can be activated through three major pathways, including the classical, lectin, and alternative lectin pathways. Complement activation, regardless of the pathway, ultimately triggers three effector pathways (MAC assembly, anaphylatoxins, and opsonization) that enable the complement to fulfill its physiological role in host defense. The lectin pathway is initiated by binding of pattern-recognition molecules, including FCN, and this pathway is accelerated by MASP, which was upregulated in pre-splenectomy serum [46]. FCN1 is a multimeric protein consisting of an N-terminal collagen-like domain and a C-terminal fibrinogen-like domain, which is present in secretory granules and is primarily expressed in granulocytes and monocytes. Serum levels of FCN1 are elevated in vasculitis syndrome and arthritis, and FCN1 has thus gained attention as a potential therapeutic target in autoimmune diseases [47]. FCN1 RNA expression was also up-regulated in the peripheral blood in dogs with IMHA [48]. In the present study, FCN1 and MASP2 were up-regulated 4- and 2.8-fold, respectively, in pre- compared with post-splenectomy serum, suggesting that uncontrolled non-regenerative anemia with complement activation may be relevant to the immune-mediated destruction of erythroid progenitor cells, which has been considered as a pathophysiology of PIMA [6]. However, FCN1 expression data could not be obtained in our transcriptome analysis, and MASP2 expression levels were similar between samples from dogs with PIMA and healthy dogs, despite their significant upregulation in serum before the splenectomy. MASP2 is reported to be produced mainly in the liver [49], and FCN1 is located primarily in peripheral blood [50, 51], suggesting that these genes are not highly expressed in the spleen, which may explain this discrepancy between the transcriptomic and proteomic data. Nevertheless, since FCN1 and MASP2 are involved in the lectin pathway that induces the activation of complement pathway [52], we speculate that the changes in levels of these proteins in the serum may be affected by the release of a specific factor, such as S100A8/A9 (Fig 5).

Considering the overall mechanism prior to splenectomy in dogs with PIMA, we predicted that S100A8/9 would be increased in the spleen in PIMA dogs, resulting in activation of the lectin pathway (Fig 5). However, the reason behind the up-regulation of S100A8/A9 in the spleen remains unknown. Expression levels of S100A8 and S100A9 in various organs have been reported to increase with ageing [53]. PIMA is most commonly reported in middle-aged and older dogs, but a previous study reported a median onset age of 6.5 years [4]. In contrast, the median age in the current group was 12 years, which was similar to a retrospective study of Miniature Dachshunds with suspected PIMA in Japan, which reported a median age of 10.9 years [54]. Despite the differences in median age compared with Assenmacher et al.’s study [4], we speculated that there might be an association between the characteristic clinical course of PIMA and the high expression of S100A8/A9 because the dogs in these three studies were older than 6.5 years.

There are several limitations to this study. Insufficient samples from both non-PIMA and healthy dog groups due to difficulty in obtaining samples. Majority of samples were MDs though this is the consistency of the previous report [4]. Pathophysiology may differ depending on treatment responsiveness to splenectomy. Further large-scale studies in specific breeds and organs and in different treatment settings are needed to identify the precise pathology of PIMA in dogs.

## Supporting information

S1 Table Clinical and histological characteristics of dogs with precursor-targeted immune-mediated anemia and healthy control dogs. CM, castrated male; F, female; M, male; MD, Miniature Dachshund; PIMA, precursor-targeted immune-mediated anemia; SF, spayed female.
(XLSX)

S2 Table All differentially expressed genes. (XLSX)

S3 Table Top 10 upregulated or downregulated pathways and genes mapped to each pathway. (XLSX)

S4 Table All proteins met the analysis criteria. (XLSX)

S5 Table Pathways of altered proteins in pre- and post-splenectomy serum samples. (XLSX)

## References

[1] Stokol T, Blue JT, French TW. Idiopathic pure red cell aplasia and nonregenerative immune-mediated anemia in dogs: 43 cases (1988–1999). J Am Vet Med Assoc. 2000;216: 1429–1436. doi: 10.2460/javma.2000.216.1429 10800515

[2] Woolhead VL, Szladovits B, Chan A, Swann JW, Glanemann B. Breed predispositions, clinical findings, and prognostic factors for death in dogs with nonregenerative immune-mediated anemia. J Vet Intern Med. 2021;35: 252–260. doi: 10.1111/jvim.15986 33617109 PMC7848385

[3] Lucidi CA, de Rezende CLE, Jutkowitz LA, Scott MA. Histologic and cytologic bone marrow findings in dogs with suspected precursor-targeted immune-mediated anemia and associated phagocytosis of erythroid precursors. Vet Clin Pathol. 2017;46: 401–415. doi: 10.1111/vcp.12502 28582594

[4] Assenmacher TD, Jutkowitz LA, Koenigshof AM, Lucidi CA, Scott MA. Clinical features of precursor-targeted immune-mediated anemia in dogs: 66 cases (2004–2013). J Am Vet Med Assoc. 2019;255: 366–376. doi: 10.2460/javma.255.3.366 31298643

[5] Akiyoshi M, Hisasue M, Neo S, Akiyoshi M. Presumptive precursor-targeted immune-mediated anemia concurrent with gastrointestinal lymphoma in a cat. J Vet Med Sci. 2020;82: 1570–1576. doi: 10.1292/jvms.20-0386 32863286 PMC7719880

[6] Lucidi CA, Gerlach JA, Jutkowitz A, Scott MA. Immunoglobulin G and phosphatidylserine in regenerative and nonregenerative immune-mediated anemias of dogs. J Vet Intern Med. 2021;35: 2713–2721. doi: 10.1111/jvim.16278 34716708 PMC8692184

[7] Akiyoshi M, Hisasue M, Neo S, Akiyoshi M. Precursor-targeted immune-mediated anemia in a dog with a stage IV mast cell tumor and bone marrow infiltration. Vet Clin Pathol. 2021;50: 151–157. doi: 10.1111/vcp.12982 33655582

[8] Bestwick JP, Skelly BJ, Swann JW, Glanemann B, Bexfield N, Gkoka Z, et al. Splenectomy in the management of primary immune-mediated hemolytic anemia and primary immune-mediated thrombocytopenia in dogs. J Vet Intern Med. 2022;36: 1267–1280. doi: 10.1111/jvim.16469 35801263 PMC9308443

[9] Akpek G, McAneny D, Weintraub L. Comparative response to splenectomy in Coombs-positive autoimmune hemolytic anemia with or without associated disease. Am J Hematol. 1999;61: 98–102. doi: 10.1002/(sici)1096-8652(199906)61:2&lt;98::aid-ajh4&gt;3.0.co;2-g 10367787

[10] Bourgeois E, Caulier MT, Delarozee C, Brouillard M, Bauters F, Fenaux P. Long-term follow-up of chronic autoimmune thrombocytopenic purpura refractory to splenectomy: a prospective analysis. Br J Haematol. 2003;120: 1079–1088. doi: 10.1046/j.1365-2141.2003.04211.x 12648082

[11] Feldman BF, Handagama P, Lubberink AA. Splenectomy as adjunctive therapy for immune-mediated thrombocytopenia and hemolytic anemia in the dog. J Am Vet Med Assoc. 1985;187: 617–619. 4086369

[12] Horgan JE, Roberts BK, Schermerhorn T. Splenectomy as an adjunctive treatment for dogs with immune-mediated hemolytic anemia: ten cases (2003–2006). J Vet Emerg Crit Care. 2009;19: 254–261. doi: 10.1111/j.1476-4431.2009.00419.x 19691510

[13] Morishita K, Sugawara M, Yamazaki J, Kim S, Hosoya K, Sasaki N, et al. Evaluation of the therapeutic efficacy of splenectomy in 20 dogs with non-regenerative immune-mediated anemia. Proceedings of College of Veterinary Internal Medicine Forum 2022; 2022 Jun 23–25; Austin, Texas. US: Wiley; 2022.

[14] Mahévas M, Patin P, Huetz F, Descatoire M, Cagnard N, Bole-Feysot C, et al. B cell depletion in immune thrombocytopenia reveals splenic long-lived plasma cells. J Clin Invest. 2013;123: 432–442. doi: 10.1172/JCI65689 23241960 PMC3533302

[15] Weiss DJ. Bone marrow pathology in dogs and cats with non-regenerative immune-mediated haemolytic anaemia and pure red cell aplasia. J Comp Pathol. 2008;138: 46–53. doi: 10.1016/j.jcpa.2007.10.001 18083185

[16] Chen S, Zhou Y, Chen Y, Gu J. fastp: an ultra-fast all-in-one FASTQ preprocessor. Bioinformatics. 2018;34: i884–i890. doi: 10.1093/bioinformatics/bty560 30423086 PMC6129281

[17] Patro R, Duggal G, Love MI, Irizarry RA, Kingsford C. Salmon provides fast and bias-aware quantification of transcript expression. Nat Methods. 2017;14: 417–419. doi: 10.1038/nmeth.4197 28263959 PMC5600148

[18] R Core Team. R: a language and environment for statistical computing. Vienna, Austria; 2011. Available from: 10.1007/978-3-540-74686-7.

[19] Barrios D, Prieto C. RJSplot: Interactive Graphs with R. Mol Inform. 2018;37. doi: 10.1002/minf.201700090 28980447

[20] Bankhead P, Loughrey MB, Fernández JA, Dombrowski Y, McArt DG, Dunne PD, et al. QuPath: Open source software for digital pathology image analysis. Sci Rep. 2017;7: 16878. doi: 10.1038/s41598-017-17204-5 29203879 PMC5715110

[21] Schmidt U, Weigert M, Broaddus C, Myers G. Cell detection with star-convex polygons. International Conference on Medical Image Computing and Computer-Assisted Intervention (MICCAI); 2018 Sep 16–20; Granada, Spain: Springer, 2018.

[22] Rappsilber J, Mann M, Ishihama Y. Protocol for micro-purification, enrichment, pre-fractionation and storage of peptides for proteomics using StageTips. Nat Protoc. 2007;2: 1896–1906. doi: 10.1038/nprot.2007.261 17703201

[23] Amodei D, Egertson J, MacLean BX, Johnson R, Merrihew GE, Keller A, et al. Improving precursor selectivity in data-independent acquisition using overlapping windows. J Am Soc Mass Spectrom. 2019;30: 669–684. doi: 10.1007/s13361-018-2122-8 30671891 PMC6445824

[24] Kawashima Y, Watanabe E, Umeyama T, Nakajima D, Hattori M, Honda K, et al. Optimization of data-independent acquisition mass spectrometry for deep and highly sensitive proteomic analysis. Int J Mol Sci. 2019;20. doi: 10.3390/ijms20235932 31779068 PMC6928715

[25] MacLean B, Tomazela DM, Shulman N, Chambers M, Finney GL, Frewen B, et al. Skyline: an open source document editor for creating and analyzing targeted proteomics experiments. Bioinformatics. 2010;26: 966–968. doi: 10.1093/bioinformatics/btq054 20147306 PMC2844992

[26] Searle BC, Swearingen KE, Barnes CA, Schmidt T, Gessulat S, Küster B, et al. Generating high quality libraries for DIA MS with empirically corrected peptide predictions. Nat Commun. 2020;11: 1548. doi: 10.1038/s41467-020-15346-1 32214105 PMC7096433

[27] Gessulat S, Schmidt T, Zolg DP, Samaras P, Schnatbaum K, Zerweck J, et al. Prosit: proteome-wide prediction of peptide tandem mass spectra by deep learning. Nat Methods. 2019;16: 509–518. doi: 10.1038/s41592-019-0426-7 31133760

[28] Searle BC, Pino LK, Egertson JD, Ting YS, Lawrence RT, MacLean BX, et al. Chromatogram libraries improve peptide detection and quantification by data independent acquisition mass spectrometry. Nat Commun. 2018;9: 5128. doi: 10.1038/s41467-018-07454-w 30510204 PMC6277451

[29] Schneider RK, Schenone M, Ferreira MV, Kramann R, Joyce CE, Hartigan C, et al. Rps14 haploinsufficiency causes a block in erythroid differentiation mediated by S100A8 and S100A9. Nat Med. 2016;22: 288–297. doi: 10.1038/nm.4047 26878232 PMC4870050

[30] Schonthaler HB, Guinea-Viniegra J, Wculek SK, Ruppen I, Ximénez-Embún P, Guío-Carrión A, et al. S100A8-S100A9 protein complex mediates psoriasis by regulating the expression of complement factor C3. Immunity. 2013;39: 1171–1181. doi: 10.1016/j.immuni.2013.11.011 24332034

[31] Holzinger D, Foell D, Kessel C. The role of S100 proteins in the pathogenesis and monitoring of autoinflammatory diseases. Mol Cell Pediatr. 2018;5: 7. doi: 10.1186/s40348-018-0085-2 30255357 PMC6156694

[32] Vogl T, Pröpper C, Hartmann M, Strey A, Strupat K, van den Bos C, et al. S100A12 is expressed exclusively by granulocytes and acts independently from MRP8 and MRP14. J Biol Chem. 1999;274: 25291–25296. doi: 10.1074/jbc.274.36.25291 10464253

[33] Ma L, Sun P, Zhang J-C, Zhang Q, Yao S-L. Proinflammatory effects of S100A8/A9 via TLR4 and RAGE signaling pathways in BV-2 microglial cells. Int J Mol Med. 2017;40: 31–38. doi: 10.3892/ijmm.2017.2987 28498464 PMC5466387

[34] Foell D, Wittkowski H, Kessel C, Lüken A, Weinhage T, Varga G, et al. Proinflammatory S100A12 can activate human monocytes via Toll-like receptor 4. Am J Respir Crit Care Med. 2013;187: 1324–1334. doi: 10.1164/rccm.201209-1602OC 23611140

[35] Wang S, Song R, Wang Z, Jing Z, Wang S, Ma J. S100A8/A9 in Inflammation. Front Immunol. 2018;9: 1298. 29942307 10.3389/fimmu.2018.01298 PMC6004386

[36] Kang KY, Woo JW, Park SH. S100A8/A9 as a biomarker for synovial inflammation and joint damage in patients with rheumatoid arthritis. Korean J Intern Med. 2014;29: 12–19. doi: 10.3904/kjim.2014.29.1.12 24574827 PMC3932383

[37] Meijer B, Gearry RB, Day AS. The role of S100A12 as a systemic marker of inflammation. Int J Inflam. 2012;2012: 907078. doi: 10.1155/2012/907078 22811950 PMC3395136

[38] Giudice V, Wu Z, Kajigaya S, Ibanez MPF, Rios O, Cheung F, et al. Circulating S100A8 and S100A9 protein levels in plasma of patients with acquired aplastic anemia and myelodysplastic syndromes. Cytokine. 2019;113: 462–465. doi: 10.1016/j.cyto.2018.06.025 29958797 PMC6336387

[39] Villa F, Malovini A, Carrizzo A, Spinelli CC, Ferrario A, Maciąg A, et al. Serum BPIFB4 levels classify health status in long-living individuals. Immun Ageing. 2015;12: 27. doi: 10.1186/s12979-015-0054-8 26675039 PMC4678610

[40] Ciaglia E, Montella F, Lopardo V, Scala P, Ferrario A, Cattaneo M, et al. Circulating BPIFB4 levels associate with and influence the abundance of reparative monocytes and macrophages in long living individuals. Front Immunol. 2020;11: 1034. doi: 10.3389/fimmu.2020.01034 32547549 PMC7272600

[41] Villa F, Carrizzo A, Spinelli CC, Ferrario A, Malovini A, Maciąg A, et al. Genetic analysis reveals a longevity-associated protein modulating endothelial function and angiogenesis. Circ Res. 2015;117: 333–345. doi: 10.1161/CIRCRESAHA.117.305875 26034043 PMC5496930

[42] Hanzawa K, Hiraga A, Yoshida Y, Hara H, Kai M, Kubo K, et al. Effects of exercise on plasma haptoglobin composition in control and splenectomized thoroughbred horses. J Equine Sci. 2002;13: 89–92.

[43] Harvey JW, West CL. Prednisone-induced increases in serum alpha-2-globulin and haptoglobin concentrations in dogs. Vet Pathol. 1987;24: 90–92. doi: 10.1177/030098588702400115 2435050

[44] McGrotty YL, Knottenbelt CM, Ramsey IK, Reid SWJ, Eckersall PD. Haptoglobin concentrations in a canine hospital population. Vet Rec. 2003;152: 562–564. doi: 10.1136/vr.152.18.562 12751608

[45] McGrotty YL, Arteaga A, Knottenbelt CM, Ramsey IK, Eckersall PD. Haptoglobin concentrations in dogs undergoing trilostane treatment for hyperadrenocorticism. Vet Clin Pathol. 2005;34: 255–258. doi: 10.1111/j.1939-165x.2005.tb00050.x 16134074

[46] Dunkelberger JR, Song WC. Complement and its role in innate and adaptive immune responses. Cell Res. 2009;20: 34–50. doi: 10.1038/cr.2009.139 20010915

[47] Katayama M, Ota K, Nagi-Miura N, Ohno N, Yabuta N, Nojima H, et al. Ficolin-1 is a promising therapeutic target for autoimmune diseases. Int Immunol. 2019;31: 23–32. doi: 10.1093/intimm/dxy056 30169661 PMC6364620

[48] Borchert C, Herman A, Roth M, Brooks AC, Friedenberg SG. RNA sequencing of whole blood in dogs with primary immune-mediated hemolytic anemia (IMHA) reveals novel insights into disease pathogenesis. PLoS One. 2020;15: e0240975. doi: 10.1371/journal.pone.0240975 33091028 PMC7580939

[49] Seyfarth J, Garred P, Madsen HO. Extra-hepatic transcription of the human mannose-binding lectin gene (mbl2) and the MBL-associated serine protease 1–3 genes. Mol Immunol. 2006;43: 962–971. doi: 10.1016/j.molimm.2005.06.033 16112196

[50] Honoré C, Rørvig S, Munthe-Fog L, Hummelshøj T, Madsen HO, Borregaard N, et al. The innate pattern recognition molecule Ficolin-1 is secreted by monocytes/macrophages and is circulating in human plasma. Mol Immunol. 2008;45: 2782–2789. doi: 10.1016/j.molimm.2008.02.005 18343499

[51] Liu Y, Endo Y, Iwaki D, Nakata M, Matsushita M, Wada I, et al. Human M-Ficolin is a secretory protein that activates the lectin complement pathway. The Journal of Immunology. 2005;175: 3150–3156. doi: 10.4049/jimmunol.175.5.3150 16116205

[52] Endo Y, Matsushita M, Fujita T. Role of ficolin in innate immunity and its molecular basis. Immunobiology. 2007;212: 371–379. doi: 10.1016/j.imbio.2006.11.014 17544822

[53] Swindell WR, Johnston A, Xing X, Little A, Robichaud P, Voorhees JJ, et al. Robust shifts in S100a9 expression with aging: A novel mechanism for chronic inflammation. Sci Rep. 2013;3: 1–13. doi: 10.1038/srep01215 23386971 PMC3564041

[54] Tani A, Tomiyasu H, Ohmi A, Ohno K, Tsujimoto H. Clinical and clinicopathological features and outcomes of Miniature Dachshunds with bone marrow disorders. J Vet Med Sci. 2020;82: 771–778. doi: 10.1292/jvms.19-0439 32307340 PMC7324823

